# Fluidic Automation of Nitrate and Nitrite Bioassays in Whole Blood by Dissolvable-Film Based Centrifugo-Pneumatic Actuation

**DOI:** 10.3390/s130911336

**Published:** 2013-08-26

**Authors:** Charles E. Nwankire, Di-Sien S. Chan, Jennifer Gaughran, Robert Burger, Robert Gorkin, Jens Ducrée

**Affiliations:** 1 Biomedical Diagnostics Institute, National Centre for Sensor Research, School of Physical Sciences, Dublin City University, Glasnevin, Dublin 9, Ireland; E-Mails: charles.nwankire@dcu.ie (C.N.); disien.chan2@mail.dcu.ie (D.C.); jennifer.gaughran3@mail.dcu.ie (J.G.); 2 Department of Micro- and Nanotechnology, Technical University of Denmark, Ørsteds Plads 345Ø, DK2800 Kgs, Lyngby, Denmark; E-Mail: robert.dcu@gmail.com; 3 Intelligent Polymer Research Institute, ARC Centre of Excellence for Electromaterials Science, AIIM Facility, Innovation Campus, University of Wollongong, Wollongong, NSW 2522, Australia; E-Mail: robertgorkiniii@gmail.com

**Keywords:** dissolvable film, sacrificial valve, reagent storage, centrifugal, microfluidics, automation

## Abstract

This paper demonstrates the full centrifugal microfluidic integration and automation of all liquid handling steps of a 7-step fluorescence-linked immunosorbent assay (FLISA) for quantifying nitrate and nitrite levels in whole blood within about 15 min. The assay protocol encompasses the extraction of metered plasma, the controlled release of sample and reagents (enzymes, co-factors and fluorescent labels), and incubation and detection steps. Flow control is implemented by a rotationally actuated dissolvable film (DF) valving scheme. In the valves, the burst pressure is primarily determined by the radial position, geometry and volume of the valve chamber and its inlet channel and can thus be individually tuned over an extraordinarily wide range of equivalent spin rates between 1,000 RPM and 5,500 RPM. Furthermore, the vapour barrier properties of the DF valves are investigated in this paper in order to further show the potential for commercially relevant on-board storage of liquid reagents during shelf-life of bioanalytical, ready-to-use discs.

## 1. Introduction

Centrifugal microfluidic platforms have proliferated significantly in the academic community and commercial world with predominant application for bioanalytical assays in biomedical diagnostics and other life science disciplines [1–5]. A prominent advantage of these “lab-on-a-disc” systems is their robust liquid handling which is widely independent of the sample properties, such as surface tension and viscosity, which typically vary to a significant extent amongst biosamples and reagents. Lab-on-a-chip platforms also excel with their comprehensive repertoire of high-performance laboratory unit operations (LUOs) for sample preparation such as sedimentation, lysis, metering, decanting, aliquoting, mixing and reagent storage [6–10]. Testing procedures such as common immunoassays consist of a multi-step sequence of such LUOs. In order to coordinate the timing of the constituent protocol steps as well as to allow on-board storage and gating of several liquids handled in parallel, a sophisticated valving strategy needs to be implemented.

The eventual choice of the valving strategy is guided by the level of process integration, automation and parallelisation that can be pursued. The simplest approach is to load reagents on demand as they are needed in the assay protocol [2,11]. The valves in this case need to function during the rather short, minute-scale processing routine, only. Nevertheless, as reagents are hard to add to disc-based inlet ports during rotation, this method implies a stop-and-go rotational protocol which would involve a number of interspersed, rather cumbersome manual pipetting steps or advanced liquid handling instrumentation. Furthermore, uncontrolled spreading of on-board liquids, e.g., through prevalent capillary action, is difficult to contain during the halt of the disc. So while this approach is feasible during development of the microfluidic chip, it lacks the user-friendliness and cost-efficiency typically imposed on point-of-care platforms.

The second level of process automation is to preload the sample and all reagents just before starting the centrifugal protocol. This scheme clearly enhances the ease of operation as, once the disc is furnished with the sample and reagents, the assay can run unattended. Also unwanted liquid spreading is suppressed as all on-board liquids may continuously be exposed to the centrifugal force. The requirements on the involved valving mechanism are somewhat enhanced compared to the previous stop-and-go protocol as the valves need to hold back reagents during the initial sample processing phase, which typically involves high-frequency centrifugation. Furthermore, this approach still leaves issues of error prone reagent logistics and system setup procedures which are undesirable in a typical point-of-care environment.

Some fully integrated and automated centrifugal platforms have been proposed over the years [3,5,12–15]. For example, Steigert *et al.* showed a fully automated assay protocol on a disc for the detection of alcohol in whole blood [5]. The authors implemented passive valving (capillary and hydrophobic stops) techniques which accurately determined the level of alcohol from a metered, 500 nL blood volume. However, while functioning in this simple assay protocol, the surface-tension based valving principle limits storage time of the disposable microfluidic cartridge and, even more, restricts the number of discrete burst frequencies required for concatenating more complex assay procedures. Serial siphoning [16], which is also a passive valving technique that involves intermittent geometrical expansions relies on hydrophilization of channels using plasma surface treatment [17] or surfactants [18] to ensure adequate priming of the channels.

Therefore active valves based on sacrificial barrier materials are increasingly employed for bioassay protocols on a disc [3,8,19]. Recently, Lee *et al.* demonstrated a fully integrated and automated centrifugal platform for the detection of Hepatitis B virus [3]. The authors implemented active ferrowax valves, which were sequentially opened using low-intensity laser light. Immunoassays and other biochemical tests from whole blood were carried out within minutes. Although this system is highly innovative, a laser is necessary to actuate the valves and wax has to be introduced into the microfluidic disposable, thus adding further complexity to the system concept.

Lately, Godino *et al.* [20] fully integrated and automated a multi-step bio-assay through a so-called centrifugo-pneumatic cascade on a lab-on-a-disc platform. Multi-liquid flow control of the sample and several pre-loaded reagents is achieved solely through rotational actuation of siphons, *i.e.*, without the need for surface modification or geometrical microfeatures. Instead, the siphons are primed by the expansion of centrifugally compressed air pocket upon reduction of the spin speed. While the centrifugo-pneumatic platform boasts very simple flow control, the open-channel structure does not provide a vapour barrier for on-disc reagent storage.

**Figure 1.**
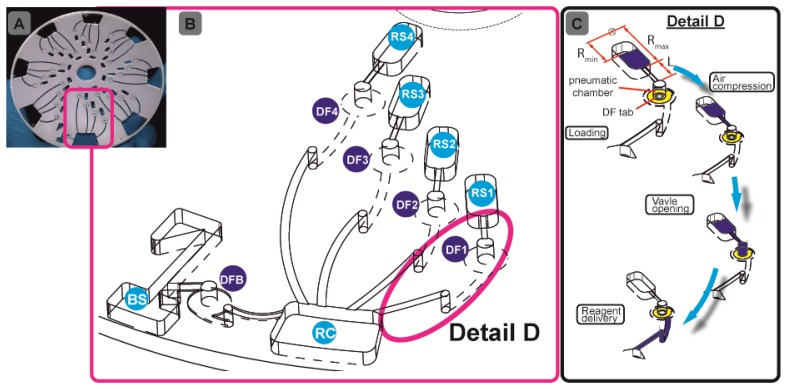
(**A**) Photo of a fully assembled nitrate and nitrite disc (∅ 120 mm; showing the eight parallel assay structures, one for the sample plus seven co-running calibration standards; (**B**) Enlarged segment of a single-assay structure featuring blood separation (BS) controlled by a DFB- (DF-blood) valve, four reagent storage chambers (RS1-RS4) controlled by DF valves DF1-DF4, respectively, and the reaction chamber (RC) as the common outlet; (**C**) Schematic of the DF valving technique with a sequence of sample loading, air compression, layer inversion/valve opening and reagent delivery through valve site.

We will here introduce a “plug-and-play” strategy which widely eliminates the need for cumbersome manual or instrumentally complex pipetting. For the first time, all assay reagents may be stored on the disc at the time of manufacture, and released on-demand during the assay run by the system-intrinsic rotational actuation (Figure 1A). This will on the one hand guarantee direct, off-the-shelf use for maximum user convenience. On the other hand, we will keep the complexity of instrumentation at a minimum as the reagents are released upon a simple change of the frequency of rotation.

Our assay integration is based on a recently developed, centrifugo-pneumatic valving technique based on water-dissolvable films (DFs) [21] that provide the physical, vapour-proof gating of sacrificial valves while being actuated through mere passive rotation. Below a critical burst pressure which scale with the square of the spinning frequency, liquid is prevented from reaching (and thus dissolving) the DF valve by a pocket of entrapped air above the DF membrane until a critical burst pressure is reached. Beyond the critical burst frequency, the metastable gas-liquid stack flips to its low-energy state, *i.e.*, the heavier liquid resides farther away from the centre of rotation than the gas. The liquid then enters the pneumatic compression chamber to contact, and hence dissolve the DF membrane and then protrude into the downstream reaction chamber (RC) (Figure 1B). Here we present a detailed investigation of the parameters governing the valve actuation mechanism to significantly extend the range of burst frequencies. This way we implement for the first time a fully integrated and automated, 7-step FLISA on the centrifugal microfluidic platform which performs blood separation and sequential release of four different reagents to measure nitrate and nitrite levels in whole blood.

Nitrate and nitrite assays have relevance in various applications. For example researchers have demonstrated the importance of nitrate and nitrite detection in blood plasma for general clinical chemistry [22,23]. It has also been shown that an elevated nitrate level in infants (less than 6 months old) could lead to shortness of breath and blue baby syndrome [24]. Shiddiky *et al.* [25] reported the electrochemical detection of nitrate and nitrite in water and urine samples towards monitoring of environmental pollution. In this work, we demonstrate the high application potential of our unique DF-based valving technique by integrating and automating off-the-shelf fluorometric nitrate and nitrite bioassays on the microfluidic disc platform, which is fully controlled by the rotational frequency.

## 2. Materials and Methods

### 2.1. Design and Fabrication of the Disc Platform

We documented the details of fabricating, assembling and embedding DF plugs in our previous work [21]. In brief, the disc consists of five layers: three layers of 1.5-mm thick poly- (methylmethacrylate) (PMMA) sheets (Radionics, Dublin Ireland) and two layers of ˜ 90 μm thick pressure sensitive adhesives (PSA) from Adhesives Research, Limerick, Ireland. A series of design iterations was assisted by CAD software. The optimised disc design with chambers and access holes was machined using a Zing CO_2_ laser cutter (Epilog Laser, Golden, CO, USA).

The chambers were tailored to the desired reagent volume. The pneumatic chambers are 4 mm in diameter. The exit channel from the blood separation (BS) chamber (Figure 1B) has been designed so as to fundamentally prevent leakage of whole blood into the pneumatic chamber. The microchannels were defined in the PSA using a Craft ROBO knife cutter (Graphtec Corp, Irvine, CA, USA). The microchannels are 0.6 mm wide. The hybrid DF valve tabs were fabricated by firstly cutting 1-mm diameter through holes in the PSA, and then rolling the ˜ 20 μm DFs (also supplied by Adhesives Research) on the PSA, in order to cut out a ∅ 3.5-mm outline. After placing the DF tabs at the designated valve locations (DFB (DF-blood;, DF1-DF4, Figure 1B) the 5-layer discs (Figure 1A) are assembled using a standard hydraulic laminator. The DF is a proprietary product of Adhesives Research.

### 2.2. Biological Assays

**Figure 2.**
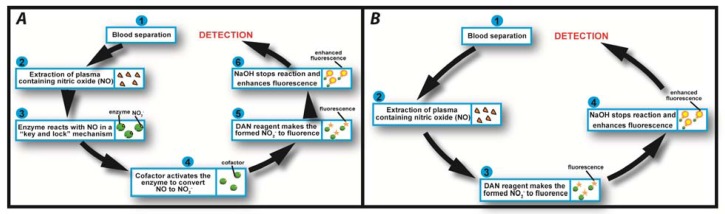
(**A**) Sequence of the homogeneous nitrate+nitrite assay (disc 1) Step 1: Separation of blood plasma; Step 2: Plasma containing nitric oxide (NO) is extracted from the blood separation chamber; Step 3: Enzyme (nitrate reductase) specifically reacts with NO in the extracted plasma; Step 4: Cofactor activates the enzyme to convert NO to NO^2−^; Step 5: DAN reagent reacts with the formed NO^2−^ to produce fluorescence and finally Step 6: NaOH is added to stop the enzymatic reaction and enhance fluorescence for off-disc read-out using a fluorescence analyser (**B**) Nitrite assay (disc 2). The steps are similar, however, without the addition of the enzyme and cofactor steps.

In this study we employed fluorometric nitrate and nitrite assay kits purchased from Cambridge Biosciences, Cambridge, UK. Nitric oxide (NO) undergoes a series of reactions with several biological fluids. The final products of NO *in vivo* are nitrate (NO^3−^) and nitrite (NO^2−^). Yet, since their proportion in whole blood is small, it is quite common in clinical chemistry to independently determine the total concentration of (NO^3−^ + NO^2−^), and subsequently NO^2−^, and then calculate the NO^3−^ concentration from their difference. Using this assay kit the respective concentrations of total (NO^3−^ + NO^2−^) and NO^2−^ were determined on two separate discs. The step-by-step sequence of the on-disc assay protocol is illustrated in Figure 2. In order to determine the total (NO^3−^ + NO^2−^) concentration; nitrate reductase was added to the extracted blood plasma, followed by the addition of an enzyme cofactor. Subsequently, an acidic solution of diaminonapthalene (DAN) was introduced to the reaction chamber, eventually producing naphthotriazole (Equation 1).


(1)
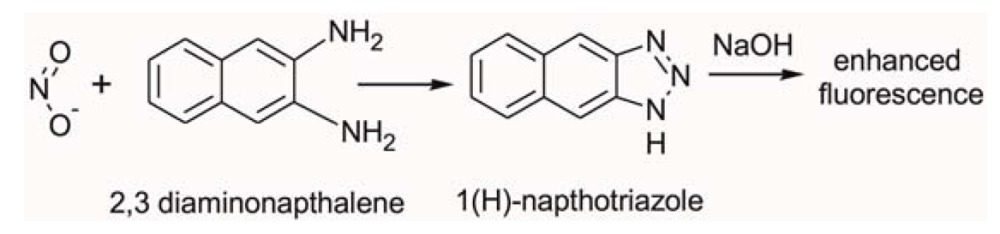


The nitrate reductase reagent converts NO^3−^ in blood plasma to NO^2−^. After this reduction reaction, the enzyme cofactor crosslinks with the formed NO^2−^ and makes it an active enzyme which is fluorescently tagged by the DAN reagent.

Following addition of NaOH solution, the enzymatic reaction stops and the fluorescence of the product (total NO^3−^ + NO^2−^) is enhanced (Figure 2A). Addition of the NaOH solution enhances the fluorescence and the analyte is ready for detection. Read-out is then performed off-chip on a fluorescence analyser at an excitation wavelength *λ* = 360–365 nm and emission at *λ* = 430 nm.

In order to determine the NO^2−^ concentration, a second disc was used. The assay procedure is similar to that of total NO^3−^ + NO^2−^ described above; however, the enzyme and cofactor were not added (Figure 2B). Fluorescence measurement of the formed product yields an accurate blood plasma NO^2−^ concentration. The NO^3−^ level was then obtained by subtracting the NO^2−^ from the total (NO^2−^ + NO^3−^) content. Calibration curves were generated according to the user manual of the commercial assay protocol.

### 2.3. Testing of the Lab-on-a-Disc Platform

The microfluidic discs are designed to concurrently run eight simultaneous tests (see Figure 1A), e.g., seven calibration standards, and one biological sample. All samples and reagents are pre-loaded on the disc and then taken to the centrifugal test stand. For disc 1, 70 μL of whole blood and assay buffer were loaded in BS and RC respectively (Figure 1B). Ten μL of nitrate reductase, enzyme cofactor and DAN reagents are loaded into RS1-RS3 and finally 20 μL of NaOH solution is loaded in RS4 (Figure 1B). The sequential release of plasma from BS and reagents from RS1-RS4 into RC is controlled by the DF valves DFB and DF1-DF4, respectively. For disc 2, 70 μL of whole blood and assay buffer were loaded in BS and RC, respectively (Figure 1B), while 10 μL of DAN reagent and NaOH were loaded in RS1 and RS2.

The centrifugal test stand, which has been fully described in previous work [21,26] comprises of a computer-controlled motor and a stroboscopic light for imaging a chosen area on the disc during rotation. The steps involved in this assay are sample metering, blood separation, plasma metering and extraction, sequential reagent delivery, mixing and, again, reagent delivery. The centrifugal force provides the pumping pressure for the liquid reagents.

### 2.4. Centrifugo-Pneumatic Actuation of the DF Valves

The normally-closed DF valve follows a centrifugo-pneumatic principle [21]. While the disc is at rest, liquid reagent in the reservoir is retained by a combination of the capillary and surface tension forces at the entrance of the microchannel leading to the dynamic formation of the pneumatic chamber. In other words, the entrance to the microchannel that connects the reservoir and the pneumatic chamber acts as a capillary stop. Rotation at the angular velocity *ω* induces a pressure head:

(2) $$\Delta p_{\omega }=\rho \bar{r}\Delta r\omega^{2}$$

(Figure 1C) where *ρ* is the liquid density, *ω* is the angular velocity of the rotating disc; *r̄* =(*R_max_* + *R_min_*)/2 is the characteristic distance of the liquid element from the centre of rotation, while Δ*r*= *R_max_* − *R_min_*, is the radial height of the liquid column. The advancing meniscus stops as Δ*p_ω_* is balanced by pressure in the valve chamber:

(3) $$p=p_{0}\frac{1}{1-\Delta V/V_{0}}$$

as obtained by Boyle's law. In Equation (3), *p*_0_ is the ambient pressure, *V*_0_ is the (full) chamber volume and Δ*V* is the gas volume reduction due to the protruding liquid meniscus [27].

The meniscus in this metastable, inverted-layer configuration is stabilized by the surface tension. As the centrifugal pressure head Δ*p_ω_* Equation (2) increases, the liquid plug can proceed farther into the compression chamber, thus making it more difficult for the surface tension to sustain the “hanging” liquid volume Δ*V*. At the so-called burst frequency, the liquid plug disrupts to invert the metastable liquid-gas configuration. Liquid then penetrates unimpeded into the pneumatic chamber to dissolve the DF and hence fully open the valve.

For a given, rotationally induced pressure head Δ*p_ω_* Equation (2), the distance the plug can protrude into the compression chamber (and the displaced air volume Δ*V*) until Δ*p_ω_* Equation (2) balances the counter pressure *p* Equation (3), which increases with the dead volume of the chamber *V*_0_. As an alternative explanation, the fluidic capacitance defined by volume change Δ*V* induced by a given pressure change *p* Equation (3) increases with *V*_0_. As the destabilisation is induced towards increasing Δ*V*, the burst frequency tends to shrink with increasing chamber volume *V*_0_.

## 3. Results and Discussion

### 3.1. Rotational Automation of Nitrate and Nitrite Bio-Assays

**Figure 3.**
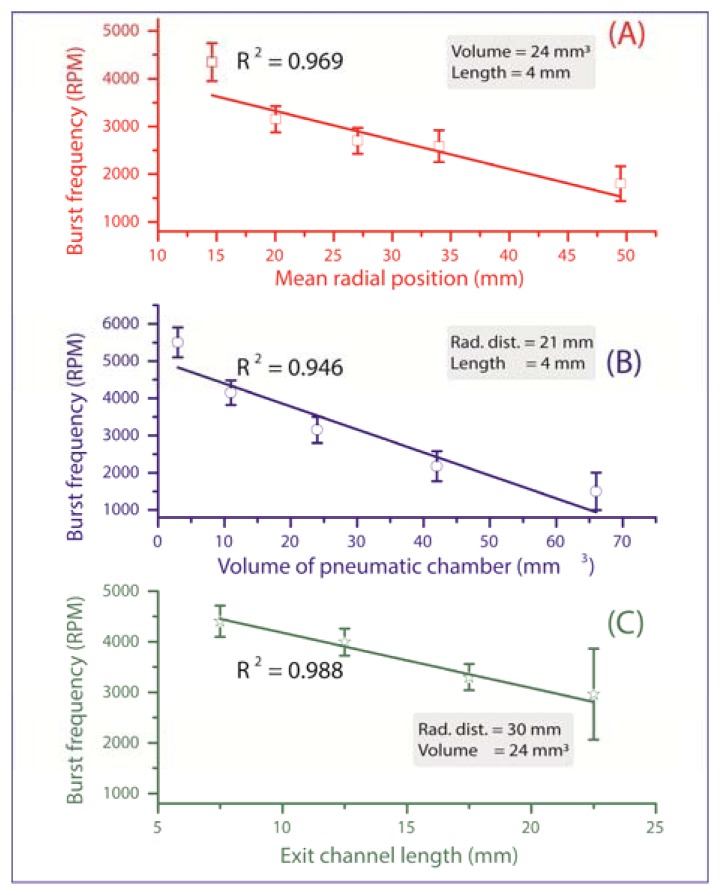
Graph demonstrating: Decrease in burst frequency as (**A**) The radial position of the DF valve on the disc platform increases, (**B**) As the volume of the compressed air increases Equation (2) and (**C**) As the exit channel length increases Equation (2). The error bars represent the standard deviation over three measurements on separate discs. The high R^2^ values of the linear fit demonstrate the predictability of this parametric study.

All valves are initially in a closed state until their individual critical burst pressure is reached, at which the valve yields and liquid is pumped onward for further processing. The measurements in Figure 3 verify the three main experimental impact parameters on the burst frequency:

(a) Mean radial position *r̄* of the liquid plug (and thus also the valve) on the disc. According to Equation (2), the centrifugal force increases with the radial distance from the centre of rotation, thus the valves which are located more radially outward are scheduled to be actuated first (Figure 3A). At a constant pneumatic chamber volume and exit channel length (see Figure 1C), when the radial position of the DF valves increased from 15 mm to 50 mm, the average centrifugal burst frequency decreased from 4,350 to 1,800 RPM. The R^2^ of the linear fit which is 0.969 demonstrate the predictability of this behaviour.
(b) Volume of the pneumatic chamber *V*_0_. According to Equation (3), the larger the volume of the compressed air, the lower the centrifugal burst pressure (Figure 3B). When the pneumatic chamber volume increased from 3 mm^3^ to 66 mm^3^ at constant exit length and radial position of the DF valves, the centrifugal burst frequency decreased from 5,500 RPM to 1,500 RPM. The R^2^ for the linear regression is 0.946.
(c) Exit channel length, L (see Figure 1C). According to Equation (2), extending the length of the liquid plug acting on the pneumatic cham­ber will decrease the burst frequency, and thus the burst pressure of the DF valve (Figure 3C). When the exit channel length increased from 7.5 mm to 22.5 mm at a constant pneumatic chamber volume and radial position of the DF valves, the centrifugal burst frequency decreased from 4,406 RPM to 2,963 RPM. The R^2^ value of the linear regression is 0.988. It should be noted that at increased channel lengths (e.g., 22.5 mm), the DF valve actuation became unstable. This instability may result from increased manufacturing tolerances (mainly in depth), leading to an undefined liquid volume entering the pneumatic chamber, thus significantly influencing the burst frequency.

**Figure 4.**
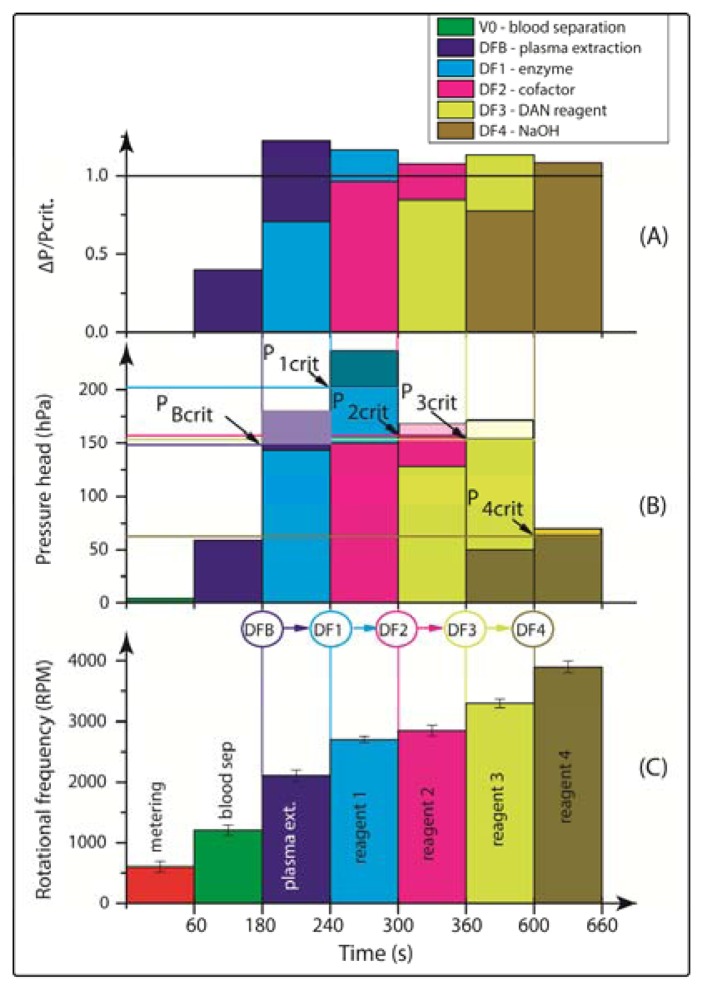
Sequential release of reagents through tuning of critical burst pressures p_crit_: (**A**) Ratio of the pressure to the critical burst pressure (**B**) Rotationally induced (absolute) pressure heads and burst pressures indicated as horizontal lines and (**C**) Corresponding rotational burst frequencies. The crossings of the same-coloured horizontal lines in (A) and (B) illustrate the sequential opening of the valves. The lighter shades of the same colour code indicate the point at which the critical burst pressures are exceeded and the corresponding valves are opened. The time axis is not drawn to scale. Raw data for this figure is provided as supplemental information.

Consequently, the rotational burst frequency as the central control parameter for all (liquid bearing) valves on the centrifugal platform can be specifically tailored over a wide range. Figure 4 for the first time demonstrates the spin protocol of a fully integrated and rotationally actuated, 7-step bio-assay protocol. Each valve is colour-coded in order to highlight the time course of the rotational burst frequency and the pressure head as deduced from Equation (2). The plot in Figure 4A references the pressure heads of each valve Equation (2) to their respective burst pressures. The sequential crossing of the horizontal (*y* = 1) -line corresponds to a valve opening, *i.e.*, the point where *p* Equation (2) exceeds the critical pressures *p_i_*_,crit_ of each valve *i*.

Figure 4A demonstrates the sequential actuation of the DF valves DFB and DF1-4 to fully integrate and rotationally automate the homogeneous nitrate and nitrite bioassays including blood separation, plasma extraction and sequential release of four different reagents. Figure 4B shows the rotationally induced (absolute) pressure heads of the liquid column as a function of the spin protocol expressed in terms of rotations per minute (RPM) displayed in Figure 4C. The critical burst pressures for the DF valves for each unit operation are indicated in the graph: plasma extraction (P_Bcrit_), DF valves 1-4 (P_1crit_ – P_4crit_). The lighter shades of this colour-coded graph indicate the points at which the critical pressures are exceeded and the DF valves are actuated. The absolute pressure head is proportional to the pressure exerted by the liquid reagent in the reservoir at its specific radial position on the disc. The first step is to meter the whole blood sample, by spinning the disc at 600 RPM for 1 min, leaving 65 μL of blood in BS chamber (Figure 1B). The disc is then rotated at 1,200 RPM for 3 min to sediment the red blood cells and separate plasma. After this plasma separation step, the rotational frequency is increased to 2,100 RPM in order to open valve #1 (DFB, Figure 1B), thus releasing 10 μL of plasma into the reaction chamber (RC), which has already been preloaded with 70 μL of assay buffer. The enzyme is released into the RC at 2,700 RPM, while the cofactor is added to the same chamber at 2,850 RPM. At this stage the assay requires an incubation step to sufficiently mix plasma + assay buffer + enzyme cofactor + nitrate reductase. To this end the disc was alternatingly spun in a clockwise and anticlockwise sense of rotation at an amplitude of 600 RPM for 3 min [12].

Upon increasing the rotational frequency to 3,300 RPM, the DAN reagent was released from reagent storage (RS3) chamber into the RC. Subsequently, NaOH was added to the same RC at a frequency of 3,900 RPM from RS4 chamber. The NaOH solution terminates the enzymatic reaction and also amplifies the fluorescence signal, as schematically illustrated in Figure 2 and Equation (1).

The procedure for the nitrite assay is very similar. In this case, only 10 μL of the DAN reagent and 20 μL of NaOH are used. The DAN reagent and NaOH solution are sequentially released into the RC while the disc rotated at 2,700 RPM and 2,900 RPM, respectively, after which 3 min of on-disc incubation is carried out by using the vigorous, “shake-mode” mixing at ±600 RPM.

**Figure 5.**
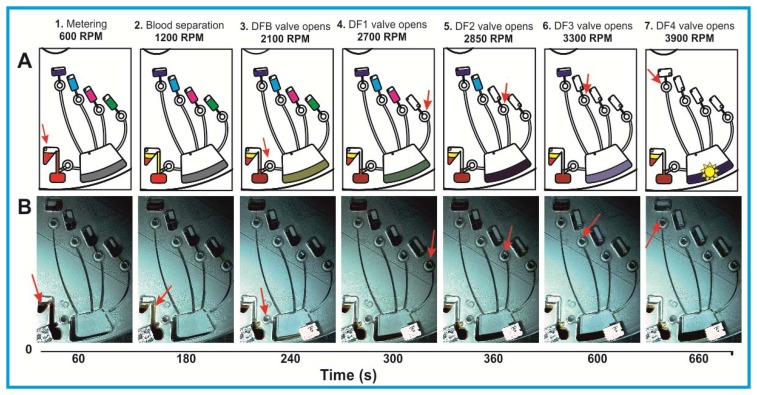
(**A**) Nitrate and nitrite assays platform-schematic of the blood plasma separation and sequential reagent delivery (**B**) Frame sequence of the DF valving technique demonstrating: (1) pre-loaded reagents and fresh whole blood sample and metering (2) blood plasma separation (3) DFB (DF-blood) valve opening and plasma extraction (5) DF1 opens after 300 s (6) DF2 valve opens after 60 s (7) Reagent mixing and incubation, followed by opening of DF3 valve opens after 600 s (8) DF4 valve actuates 60 s later, after which sample detection is carried out. The red arrow indicates the step sequence of the assay and the positions of the DF valves that will be actuated to subsequently deliver the reagent in the corresponding chamber. The full length video can be found in *ESI*. The loading hole for the assay buffer was sealed with foil to prevent leakage. This access hole was also used to collect the liquid for off-chip read-out.

A schematic representation of the liquid handling procedure and the image frame time sequence of this process are detailed in Figure 5. In order to enhance the contrast for this demonstration, coloured food dye was used. The full video of this centrifugally automated bioassay with all assay reagents including blood separation and plasma extraction processes can be found in *ESI*.

### 3.2. Assay Results

As this paper focuses on the fluidic automation aspect of a multi-step assay protocol, we decided to leave separate issue of the integration of an optical detector onto the instrument to a later stage. So for now, a 2-μL sample of the end product is taken from the disc-based reaction chamber, placed on the NanoDrop 3300 (Thermo Fisher Scientific Inc., Dublin, Ireland) pedestal for off-chip analysis.

**Figure 6.**
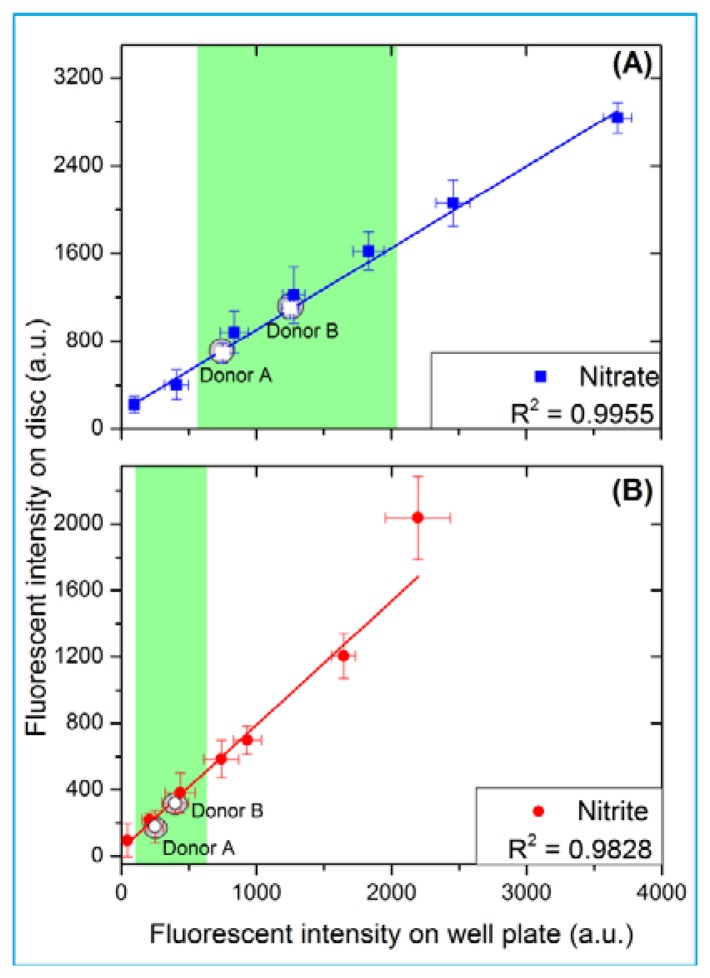
Calibration curves for (**A**) Nitrate and (**B**) Nitrite assays obtained from a standard 96-well plate and the automated disc platform showing the good correlation (R^2^ = 0.9955 and R^2^ = 0.9828, respectively) between both results. The highlighted region is the normal range of nitrate and nitrite levels for adults, and the results obtained using this assay from two anonymous blood donors (A & B) are also indicated.

As shown in Figure 6, the unknown concentration of nitrate and nitrite in the blood of two anonymous donors has been determined. A fresh whole blood sample was obtained and used on the same day. The control assays using the donated blood were initially carried out on a 96-well plate; and later implemented on the automated disc platform (Figure 6). For blood donor A, the levels of nitrate and nitrite are 20.7 μM and 8.7 μM, respectively. For blood donor B; the nitrate and nitrite levels are 10.7 μM and 5.4 μM, respectively. These levels of nitrate and nitrite are well within the normal reported clinically relevant range of 4–45.4 μM for nitrate and 1.3–13 μM for nitrite [27]. Figure 6 compares the results obtained from the 96-well plate and the automated centrifugal platform. As further shown in Figure 6; the high R^2^ of the linear regression indicates a good correlation between the data obtained from the standard well plate and that obtained from this automated disc platform.

### 3.3. Reagent Storage with the DF Valves

**Figure 7.**
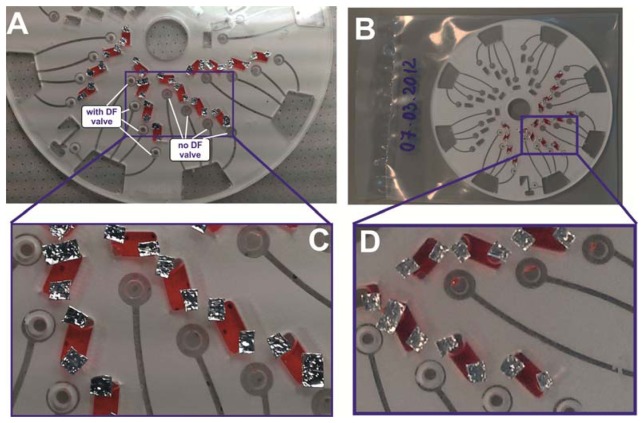
(**A**) Image of the microfluidic disc pre-loaded with red food dye, stored in a heat sealed bag in the fridge at ˜4 °C for 7 days. (**B**) Image of the disc after storage. (**C**) Enlarged image of the section with and without DF tabs before storage. (**D**) Enlarged image of the same section after 7 days of storage. Note that liquid has leaked through the channels to the pneumatic chamber region in the section without the DF valve; however, no liquid was observed at the exit channels of the chambers with the DF valve in place. The loading holes access holes used to collect the liquid for off-chip read-out were sealed with aluminium foil tape to control for loss of liquid by evaporation

In order to validate the potential of the DF barriers for longer-term reagent storage, 30 μL of coloured water were loaded in the reagent chambers; the discs were enclosed in a heat seal bag and stored in the fridge at ˜ 4 °C for a period of 7 days (see Figure 7). The aim is to assess the integrity of the valving technique after this storage period. After 7 days of storage, the disc was taken out, examined and rotated on the spin stand. It was observed that liquid droplets had leaked into the pneumatic chamber of the section of the disc without the centrifugo-pneumatic DF valves, while the sections with DF valves remained sealed. This observation provides evidence that the DF valves provided enough vapour barriers to prevent reagents from creeping into the pneumatic chamber by capillary movement.

## 4. Conclusion and Outlook

Leveraged by the broad range and sharp definition of burst frequencies, we have for the first time integrated and automated a comprehensive, 7-step bioassay protocol including plasma separation and the sequential release of four on-board stored liquid reagents by our new and only rotationally actuated DF-based centrifugo-pneumatic valving scheme. We experimentally verified that the DF valves yield at a well-defined burst pressure which can be tailored over a wide range of (equivalent) spin rates (ca. 1,000 RPM to 5,000 RPM) by the radial position of the valve on the disc, the volume of the entrapped air in the pneumatic compression chamber and the geometry of their inlet channel.

As a pilot study, we chose the detection of nitrate and nitrite starting at clinically relevant concentrations in whole blood. The results were in good quantitative agreement with the same assay carried out on a regular microtiter plate.

Regarding the potential use of DF valves for on-board storage of liquid reagents, we successfully confirmed the vapour-barrier properties of the DF valves. Future research will be directed towards integrating and parallelizing a repertoire of multi-step assay protocols based on this rotationally controlled centrifugal valving scheme. We also seek to further extend the long-term stability of the DF valves by optimizing their formulation or by applying common (surface) treatments.
